# New urinary biomarkers for diabetic kidney disease

**DOI:** 10.1186/2050-7771-1-9

**Published:** 2013-02-04

**Authors:** Cheng Wang, CuiCui Li, WenYu Gong, Tanqi Lou

**Affiliations:** 1 Division of Nephrology, Department of Medicine, Third Affiliated Hospital of Sun Yat-Sen University, Guangzhou, Guangdong, 510630, China

**Keywords:** Diabetic kidney disease, Biomarker, Urine

## Abstract

Diabetic kidney disease is the leading cause of end-stage renal disease in developed and developing countries. Microalbuminuria is the gold standard for detection and prediction of diabetic kidney disease and cardiovascular risk disease in clinical practice. However, microalbuminuria has several limitations, such as lower sensitive, larger variability. It is urgent to explore higher sensitivity and specificity for earlier detection of diabetic kidney disease and more accurate prediction of the progression to end stage renal disease. We reviewed some new and important urinary biomarkers, such as: transferrin, immunoglobulin G, immunoglobulin M, Cystanic C, podocytes, type IV collagen, 8-oxo-7, 8-dihydro-2'-deoxyguanosine, ceruloplasmin, monocyte chemoattractant protein-1 and so on. We need good quality, long-term, large longitudinal trials to validate published biomarkers and find new biomarkers, considering biomarkers reviewed here are from small cross-sectional studies.

## 

Diabetes mellitus (DM) is a chronic disease that affects 366 million people worldwide (6.4% of the adult population) and is expected to rise to 552 million by 2030 [1]. Diabetic kidney disease (DKD) is one of the most serious microvascular complications, which significantly impacts morbidity, mortality and quality of life. DKD occurs in approximately one-third of all people with diabetes and is the leading cause of renal failure in developed and developing countries. The first sign of DKD is considered to be microalbuminuria in clinical practice, while microalbuminuria has several limitations such as lower sensitive and larger variability. Therefore, earlier, more sensitive and specific biomarkers with greater predictability are needed. The aim of this review is to summarize new urinary biomarkers for glomerular injury associated with DKD.

## Transferrin

Transferrin, a plasma protein, is very similar to albumin in weight. It is more readily filtered through glomerular barrier than albumin for being less anionic. Urinary transferrin is considered to be a more sensitive marker of glomerular damage in diabetic patients based on theory analysis and experimental results. Urinary transferrin excretion shows a good linear relationship with urinary albumin excretion in diabetic patients, and increased urinary transferrin excretion predicts the development of microalbuminuria in type 2 diabetic patients with normoalbuminuria [2]. A systemic review, including 13 studies, indicated that urinary transferrin excretion was a good marker for predicting onset of nephropathy [3]. However, urinary transferrin excretion is not specific for DKD because its elevation can be found in primary glomerulonephritis [4].

## Immunoglobulin G

Immunoglobulin G (IgG) is a protein synthesized and secreted by plasma cells. It has a molecular weight of 150 kDa, which is larger than albumin. Urinary IgG excretion is higher in diabetic patients compared to healthy controls, and its excretion in diabetic patients with normoalbuminuria predicts the development of microalbuminuria [5]. Urinary IgG excretion correlates with the progression of glomerular diffuse lesions. One IgG isoform (IgG4) has been used more specifically as a marker of glomerular charge selectivity impairment. Only IgG4 excretion is elevated in patients with microalbuminuria, while the excretion of both IgG and IgG4 are increased in patients with macroalbuminuria compared with normoalbuminuric patients [6]. Recently, one study found that urinary concentration of IgG2 in patients with normoalbuminuria was significantly higher than in healthy control, whereas further elevation of IgG2, IgG4, and IgA was more pronounced in patients with microalbuminuria. Fractional excretion of IgG2 was the highest among all immunogloubins, which indicated that elevation of those particular immunogloubin subtypes was a contribution of novel mechanisms in early DKD, different from charge and size barrier impairment [7]. One systemic review, including 13 studies, indicated urinay IgG was a good marker for predicting onset of nephropathy [3].

## Immunoglobulin M

Immunoglobulin M (IgM), secreted by plasma cells, is the largest antibody in the human. Due to its large molecular radius, the appearance of IgM in urine indicates that a large, nonselective pore exists in the glomerular capillary wall. One study showed that urine excretion of IgM was significantly higher in type 2 DM compared to type 1 DM, and patients with type 2 DM with nephrosclerosis had significantly higher urine excretion of IgM compared to the age-matched healthy subjects [8]. Another study found renal survival of type 2 diabetic patients was inversely associated with urine IgM excretion, which indicated that higher urinary IgM excretion was a better predictor of decline in kidney function than albuminuria in type 2 DM. However, urinary IgM excretion has not been regarded as an early marker of DKD, since its excretion in urine is associated with severe injury of the glomerular capillary wall, while it is also a promising marker which may predict the eventual need for renal replacement therapy [9].

## Cystatin C

Cystatin C, a cysteine protease inhibitor, is a novel biomarker of renal damage. Serum Cystatin c is a good marker for assessing renal injuries, while urinary cystatin c was considered as a useful marker for the detection of DKD. One study from Zucker diabetic fatty (ZDF) rats indicated that urinary cystatin C was increased in ZDF rats where renal damage was not observed by histopathological assessment, and its levels in urine increased with the progression of renal damage, demonstrating the usefulness of early detection and accurate assessment of DKD [10]. Another study from type 2 diabetic patients found that urinary cystatin C increased with increasing degree of albuminuria and reached higher levels in macroalbuminuric patients. Urinary cystatin C levels were identified as an independent factor associated with estimated glomerular filtration rate (eGFR) < 60ml/min/1.73m^2^ in patients with normoalbuminuria, which indicated urinary cystatin C levels could be a useful marker for renal dysfunction in type 2 diabetic patients with normoalbuminuria [11].

## Podocytes

Podocytes are key structural elements of the glomerular filtration barrier. It is accepted that podocytes’ injuries play an essential role in the progression of DKD [12]. Monitoring urine podocytes and podocyte-specific proteins can reveal potentially interesting urinary markers for the early diagnosis of DKD [13]. Podocytes in urine can be found in diabetic patients with micro- and macroalbuminuria [14]. Another study indicated that nephrinuria was found to be present in 100% of diabetic patients with micro- and macroalbuminuria, as well as 54% of patients with normoalbuminuria; what’s more, nephrinuria also correlated positively with albuminuria, which suggested that nephrinuria might be a biomarker of early DKD [15]. Urinary podocalyxin was higher in 53.8% patients at the normoalbuminuric stage, 64.7% at the microalbuminuric stage and 66.7% at the macroalbuminuric stage, which indicated that urinary podocalyxin might be a useful biomarker for detecting early podocyte injury in diabetic patients [16]. Another study found that urinary mRNA profiles of synaptopodin, podocalyxin, α-actin-4, and podocin were increased with the progression of DKD, which suggested that quantification of podocyte-associated molecules in urine will be a useful biomarker of DKD [17].

## Type IV Collagen

Type IV collagen is the main constituent of both glomerular and tubular basement membranes as well as mesangial matrix. Urinary type IV collagen was significantly increased in both normoalbuminuric and microalbuminuric patients of type 2 DM compared with healthy controls, and urinary type IV collagen significantly correlated with the amount of albuminuria [18]. Another study found that urinary type IV collagen was more sensitive than albuminuria to detect renal damage in type 2 diabetic patients. A follow-up study showed that 25% of normoalbuminuric patients with increased urinary type IV collagen excretion developed microalbuminuria, while patients who stayed normoalbuminuria had a significant decrease in urinary type IV collagen excretion, which suggested that urinary type IV collagen is a marker to detect the progression of DKD [19,20].

## 8-oxo-7, 8-dihydro-2'-deoxyguanosine

It is well known that increased oxidative stress in diabetes contributes to the progression of diabetes and its complications. 8-oxo-7, 8-dihydro-2'-deoxyguanosine (8-oxodG), a marker of intracellular oxidative stress, can be assessed non-invasively in urine. Patients with higher excretion of 8-oxodG in urine compared with those patients with moderate or lower excretion of 8-oxodG showed significant progression of diabetic nephropathy, which indicates that 8-oxodG in urine is a useful clinical marker to predict the development of diabetic nephropathy [21,22].

## Ceruloplasmin

Ceruloplasmin (molecular weight =151 kD) is the major copper-carrying protein in blood and more negatively charged than albumin, which makes it difficult to be filtered by the glomerulus. Urinary ceruloplasmin was found in normoalbuminuric diabetic patients, and its increase in urine had a predictive value for development of microalbuminuria in normoalbuminuric diabetic patients [23,24]. The ceruloplasmin/creatinine ratio is higher in DKD compared with non-diabetic patients, and its ratio has a sensitivity of 90-91%, specificity of 61–66% in diagnosing DKD [25]. All these data suggest that urinary ceruloplasmin is a promising marker of DKD, while further studies are needed to characterize its value compared to albuminuria, especially in type 1 diabetics, since all the studies have been done in type 2 diabetics.

## Monocyte chemoattractant protein-1(MCP-1)

Chemokines have been implicated in the pathogenesis of DKD, therefore, measurement of cytokine in urine might help to diagnosis DKD. Urinary MCP-1/creatinine in patients with macroalbuminuria was significantly higher than patients with normoalbuminuria and microalbuminuria, and urinary MCP-1 correlated with the rate of eGFR decline [26]. Another study found that urinary MCP-1 was significantly higher in patients presenting doubling of serum creatinine or death, and its levels were positively associated with the risk of doubling of serum creatinine or death after Cox regression; what’s more, urinary MCP-1 remained as significant independent predictors of doubling of serum creatinine or death [27]. All these data suggested that urinary MCP-1 might be a prognostic marker for progression of diabetic nephropathy, while more studies are needed to investigate whether urinary MCP-1 has a role in the setting of normoalbuminuria and microalbuminuria in DKD.

## Neutrophil gelatinase-associated lipocalin

Neutrophil gelatinase-associated lipocalin (NGAL) has been evaluated in several studies of diabetic subjects. In one study, urine NGAL was 5–10-fold higher in normo- or microalbuminuric patients compared with healthy controls. Another study from short-term type 2 DM patients indicated urinary NGAL showed a negative correlation with eGFR, which suggested urinary NGAL might be a promising early marker for monitoring renal impairment in short-term T2DM patients [28]. Study from type 1 DM indicated that urine NGAL levels correlated with albumin/creatinine, and patients with higher albuminuria had higher urine NGAL levels, which suggested that elevated urinary NGAL values might indicate kidney damage [29].

## Identification of urinary markers by proteomic approaches

Proteomics is a method aimed at discovering and identifying the complete set of proteins present in a given biological sample at a given time. Using a variant of two dimensional gel electrophoresis, they found urine samples from type 2 DM patients with microalbuminuria showed four main proteins accompanying with albumin: alpha-2 glycoprotein, alpha-1 acid glycoprotein, alpha-1 microglobulin and IgG [30]. Otu found a 12-peak proteomic signature in the baseline urine of type 2 DM patients who subsequently developed DKD [31]. The reported accuracy (71% sensitivity and 76% specificity) is encouraging in view of a future diagnostic assay. Zürbig used capillary electrophoresis-coupled mass spectrometry to profile the low-molecular weight proteome in urine samples from a longitudinal cohort of type 1 and 2 diabetic patients. They found collagen fragments were prominent biomarkers before onset of macroalbuminuria, and there is a decrease in collagen fragments before albumin excretion starts to increase [32]. Urinary proteomics enables noninvasive assessment of DN risk at an early stage, while more studies are needed to investigate the role of urinary proteomics in diabetic kidney disease.

## Novel biomarkers

Recently, a study from uni-nephrectomized diabetic rats indicated urinary osteopontin, heart-type fatty acid binding protein appeared before the classical biomarkers of diabetic nephropathy, such as albuminuria and urinary protein excretion [33]. Study of males with Type 2 diabetes indicated human zinc-α (2) -glycoprotein might be a novel urinary biomarker for non-albuminuric diabetic nephropathy [34]. Another study suggested urinary mRNA levels of α-smooth muscle actin, fibronectin and matrix metalloproteinase-9 might be novel biomarkers of diabetic kidney disease [35]. McKittrick reported that urinary matrix metalloproteinase activity might be a sensitive, noninvasive, and clinically useful biomarker for predicting vascular remodeling in diabetic renal and vascular complications [36]. The above mentioned results are from small patient population and from animal experiments, which lead to limited use for clinical practice. We need larger perspective studies to confirm the utility of these biomarkers in diabetic kidney disease

## Conclusion and future directions

The current gold standard for detection and prediction of DKD is microalbuminuria; however, it has several limitations, such as lower sensitive and larger variability. It is urgent to explore higher sensitivity and specificity for earlier detection of DKD and more accurate prediction of the progression to end stage renal disease. Despite numbers of new biomarkers described, most studies are limited by either their small sample size or their cross-sectional nature. We need large, prospective, multi-center trials enlisting both Type 1 and Type II diabetic patients with and without nephropathy for at least two decades to indentify there role in clinical practice.

## Abbreviations

DM: Diabetes mellitus; DKD: Diabetic kidney disease; IgG: Immunoglobulin G; IgM: Immunoglobulin M; eGFR: Estimated glomerular filtration rate; 8-oxodG: 8-oxo-7, 8-dihydro-2'-deoxyguanosine; MCP-1: Monocyte chemoattractant protein-1; NGAL: Neutrophil gelatinase-associated lipocalin.

## Competing interest

We declare that we have no competing interests.

## Authors’ contributions

The review was designed by CW and TQL. CCL and WYG prepared some papers. The review was written by CW and TQL, edited by all authors, who have approved the final version.
